# Reduced contribution of thermally labile sugar lesions to DNA double strand break formation after exposure to heavy ions

**DOI:** 10.1186/1748-717X-8-77

**Published:** 2013-04-02

**Authors:** Satyendra K Singh, Alena Bencsik-Theilen, Emil Mladenov, Burkhard Jakob, Gisela Taucher-Scholz, George Iliakis

**Affiliations:** 1 Institute of Medical Radiation Biology, University of Duisburg-Essen Medical School, Hufelandstr 55, Essen, 45122, Germany; 2 GSI Helmholtzzentrum für Schwerionenforschung GmbH, Division of Biophysics, Darmstadt, Germany

**Keywords:** DNA double strand breaks (DSB), Ionizing radiation (IR), High LET, Heavy ions, Labile lesions, Radiation chemistry

## Abstract

In cells exposed to low linear energy transfer (LET) ionizing-radiation (IR), double-strand-breaks (DSBs) form within clustered-damage-sites (CDSs) from lesions disrupting the DNA sugar-phosphate backbone. It is commonly assumed that all DSBs form promptly and are immediately detected by the cellular DNA-damage-response (DDR) apparatus. However, there is evidence that the pool of DSBs detected by physical methods, such as pulsed-field gel electrophoresis (PFGE), comprises not only promptly forming DSBs (prDSBs) but also DSBs developing during lysis at high temperatures from thermally-labile sugar-lesions (TLSLs). We recently demonstrated that conversion of TLSLs to DNA breaks and ultimately to DSBs also occurs in cells during the first hour of post-irradiation incubation at physiological temperatures. Thus, TLSL-dependent DSBs (tlDSBs) are not an avoidable technique-related artifact, but a reality the cell always faces. The biological consequences of tlDSBs and the dependence of their formation on LET require in-depth investigation. Heavy-ions (HI) are a promising high-LET radiation modality used in cancer treatment. HI are also encountered in space and generate serious radiation protection problems to prolonged space missions. Here, we study, therefore, the effect of HI on the yields of tlDSBs and prDSBs. We report a reduction in the yield of tlDBSs stronger than that earlier reported for neutrons, and with pronounced cell line dependence. We conclude that with increasing LET the complexity of CDSs increases resulting in a commensurate increase in the yield prDSBs and a decrease in tlDSBs. The consequences of these effects to the relative biological effectiveness are discussed.

## Introduction

Ionizing radiation (IR) deposits energy as single ionizations or as ionization clusters that generate base and sugar damages in the DNA [1-3]. Clusters of ionization can generate clusters of DNA damage with different sizes and diverse damage composition (clustered damage sites, CDSs). Sugar damage can disrupt the sugar-phosphate backbone to generate DNA single-strand-breaks (SSBs) [2-4]. SSBs within CDSs form DNA double-strand-breaks (DSBs), which can have severe biological consequences [1,4-8].

DSBs can also be generated from CDSs populated with base damages through bi-stranded enzymatic opening during repair of the DNA sugar-phosphate backbone, or by combining with a SSB [7].

DSBs initiate rapid signaling and complex regulatory processes affecting DNA repair, cell cycle progression, transcription, translation, as well as decisions of programmed cell death and autophagy. These responses are currently integrated under the term cellular DNA-damage-response (DDR) [9].

Analysis of DDR after IR is based on the assumption that all DSBs form promptly. However, irradiation of plasmid DNA has shown that IR induces, in addition to sugar lesions promptly disrupting the sugar-phosphate backbone (prompt breaks), also lesions doing so after temperature-dependent chemical processing (delayed breaks) [10]. These thermally labile sugar lesions, TLSLs, constitute what are considered radiation-induced labile sites [1,6,10]. They can include diverse forms of sugar damage, abasic sites, and forms of base damage affecting sugar stability. Chemical evolution of such TLSLs to SSBs within a CDS can generate additional, TLSL-dependent DSBs (tlDSBs) [3,5,10-13].

Until recently, it was believed that in mammalian cells evolution of TLSLs to SSBs and the generation in this way of DSBs is only possible at high, non-physiological temperatures (~ 50°C) [14-16]. However, recent work from our laboratory [17,18] provides evidence that IR induces TLSLs, which evolve within about 1 h under physiological temperatures to SSBs and contribute, when present within a CDS, to the formation of DSBs. These delayed-forming DSBs are thought to be generated continuously during the first postirradiation hour and to add to DSBs promptly induced (prDSBs). The biological consequences of tlDSBs remain to be elucidated, but are likely to be significant.

Since tlDBSs form within CDSs, the quality and quantity of which strongly depends on the linear energy transfer (LET) of the radiation employed, it is particularly important to study determinants of formation and rules of chemical tlDSBs processing after exposure to high LET radiation. First experiments along these lines using neutrons [19] showed a marked decrease in the yield of tlDBSs.

Heavy-ions (HI) are a promising high-LET radiation modality increasingly used in cancer treatment. Carbon ions are being used for the treatment of several types of solid cancers with promising results, and advanced treatment centers with ion accelerators are under construction in several countries. Important research issues related to the biological effects of HI and their relevance to the clinical application have been identified for in-depth investigation [20]. The nature of the DNA damage induced by HI and the contribution of tlDSBs to the biological effect is one such fundamental question.

HI are also encountered in space and generate serious radiation protection problems for long-duration missions to the earth’s moon, or to Mars [21]. In preparing for such missions, the risk of cancer from space radiation must be estimated and mitigating measures must be developed. HI produce distinct forms of biological damage with largely unknown cancer risks. HI are therefore likely to require countermeasures different from those developed for low LET radiation [21]. Characterization of differences in the form of DNA damage generated by HI, particularly in the form of prDSBs and tlDSBs, is the first important step in this endeavor.

Here, we study the yields of tlDSBs and prDSBs in cells exposed to HI. We report a reduction in the yield of tlDBSs stronger than that earlier reported for neutrons [19], but still with pronounced cell line dependence.

## Materials and methods

### Cell lines and culture conditions

*M059K*, a repair proficient human glioma cell line and *M059J* its DNA-PKcs deficient counterpart [22], were grown in Dulbecco’s Modified Eagle’s Medium (D-MEM) supplemented with 10% fetal bovine serum (FBS), 1% non-essential amino acids and 1% L-glutamine. Mouse embryonic fibroblasts (MEFs) from *Lig4*^-/-^/*p53*^-/-^ and *Lig4*^+/+^/*p53*^-/-^ mice [23] were grown in D-MEM supplemented with 10% FBS and antibiotics. For experiments, cells were maintained in the exponential phase of growth at 37°C in a humidified incubator, in an atmosphere of 5% CO_2_ and 95% air.

### Irradiation conditions for heavy ions (^58^Fe) and X-rays

To analyze induction of DSBs, cells were resuspended in serum-free medium, and processed for PFGE as described earlier [17,18].

Exposures to heavy ions (HI) were carried out at the GSI Helmholtzzentrum für Schwerionenforschung GmbH in Darmstadt, Germany. Typically cells were seeded in 25 cm^2^ tissue culture flasks and were incubated for 24 h at 37°C in Essen. The following day cells were transported in an insulated container filled with warm pads to maintain the temperature of the cells as close as possible to 37°C, but without active heating. Upon arrival at the GSI, cells were promptly incubated at 37°C under standard growth conditions, and were allowed to recover for several hours from the transportation stress.

Cells were exposed to 1 GeV/amu heavy ions (^58^Fe or ^62^Ni). The particle LET under these conditions is 150 keV/μm and 175 keV/μm for ^58^Fe and ^62^Ni, respectively. Dosimetry was carried out with a calibrated farmer chamber (PTW, Freiburg, Germany). The absolute particle fluence was measured with a calibrated ionization chamber (GSI, Darmstadt, Germany) at the beam exit window and the homogeneity of the scanned field was regularly checked using radiochromic EBT films (Ashland,Gafchromic, USA). The irradiation field was 5 x 8 cm and was generated by multiple scanning of a pencil beam across the field with a dose deposition of 1 Gy per single scan. During irradiation cells were maintained at 4-8 °C. Before exposure to heavy ions, tissue culture flasks were filled with growth media and were pre-cooled in ice-water for 15 min before placement in the irradiation holder. During the actual exposure to radiation, cells were not actively cooled. After radiation exposure, cells were transferred to the laboratory in ice. Where appropriate, irradiated cells were transferred to pre-warmed (45°C) media to quickly regenerate 37°C and start repair processes. The limited availability of HI for biological experiments made repeat-experiments impossible.

Control experiments were carried out by exposing cells to X-rays. In this case, irradiations were carried out also on ice with a Seifert-Pantak X-ray machine operated at 320 kV, 10 mA with a 1.65 mm Al filter (effective photon energy, 90 keV), at a dose rate of 3 Gy/min and a distance of 50 cm. Dosimetry was carried out using a calibrated ionization chamber and a chemical dosimeter. The mean LET of this type of radiation is, approximately, 2 keV/μm.

### Pulsed-field gel electrophoresis (PFGE)

In certain experiments, cells were lysed before irradiation using the low temperature lysis (LTL) protocol described below [15] and were incubated in TEN buffer (10 mM Tris–HCl, pH 7.5, 2 mM EDTA, 50 mM NaCl) to analyze TLSL-evolution. In other experiments, cells embedded in agarose blocks were irradiated in serum-free medium and were subsequently lysed by LTL.

In the standard, high temperature lysis (HTL) protocol [24], agarose blocks were placed in lysis buffer (10 mM Tris–HCl, pH 7.6, 50 mM NaCl, 10 mM EDTA, 2% N-lauryl sarcosyl, NLS, and 0.2 mg/ml protease added just before use) at 4°C and were further processed at 50°C as described earlier [17,18]. Low temperature lysis (LTL) was carried out by maintaining samples below 4°C at all times using a published protocol [15], as described earlier [17,18].

Asymmetric, field-inversion gel electrophoresis (AFIGE) was carried out in gels cast with 0.5% molecular biology grade agarose (Bio-Rad) in the presence of 0.5 μg/ml ethidium bromide as described [17,18]. Gels were scanned using the "Typhoon" (GE-Healthcare) and the fraction of DNA released (FDR) from the well into the lane was quantified from images obtained using Image Quant 5.2 (GE-Healthcare).

### Treatment for the development of in-vitro of DSBs from TLSLs

To monitor the kinetics of excess DSB formation at different temperatures *in vitro*, cells embedded in agarose blocks were exposed to IR, subjected to LTL, washed once for 1 h in TEN buffer (0.5 ml/plug) and the resulting agarose blocks containing the “naked” DNA were distributed to different tubes in the same buffer. Tubes were then transferred to water baths adjusted at different temperatures ranging between 10°C - 50°C and incubated for different periods of time before washing once in 0.5X TBE and processing for PFGE.

## Results

### No detectable tlDSBs in some cell lines after exposure to HI

**Figure 1:**
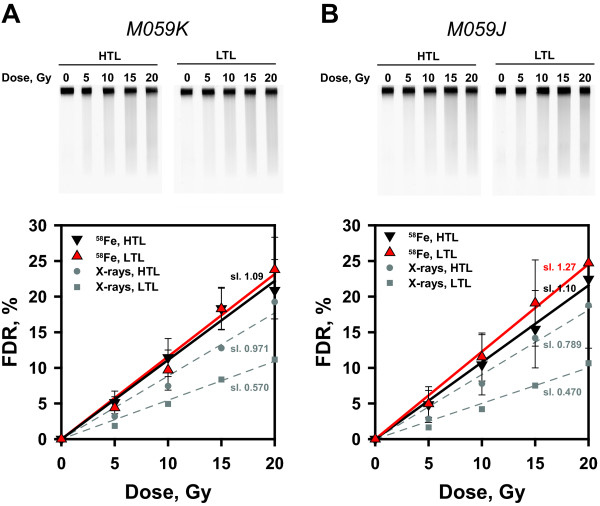
**Dose response curves for the induction of DNA DSBs after **^**58**^**Fe ion irradiation in human M059K and M059J cells.** Results earlier generated [19] with X-rays are depicted as gray symbols for comparison; they are similar to results generated in parallel to the ^58^Fe irradiations but for limited number of doses in the range of interest (not shown). (**A**) Human M059K cells were embedded in agarose blocks and were exposed to different doses of 1 GeV accelerated ^58^Fe ions. Induction of DSBs was assayed by asymmetric field inversion gel electrophoresis (AFIGE), a pulsed-field gel electrophoresis method. Irradiated agarose blocks were processed immediately after irradiation using either HTL or LTL. Gels were scanned in a Fluor Imager and the fraction of DNA released from the wells into the lane (FDR) was determined for different radiation doses. Data represent the calculated average and standard deviation from 4 determinations (agarose blocks) in one experiment. (**B**) M059J cells embedded in agarose blocks were irradiated and analyzed as described above. Data represent the calculated average and standard deviation from 4 determinations in one experiment.

Previous work suggested that the contribution of TLSLs to the overall cellular DSB load decreases with increasing LET [19]. We inquired whether this trend persists with increasing LET of the radiation employed, as is the case for HI exposures. Figure 1A summarizes results obtained after exposure of the human tumor cell line, M059K, to iron ions (^58^Fe). As previously reported [17,18], exposure at 4°C of agarose-embedded M059K cells to different X-ray doses gives after lysis with the standard HTL protocol over 40% additional DSBs than lysis with the TLSL-preserving LTL protocol; these extra DSBs (tlDSBs) are generated by the thermal conversion within a CDS of TLSLs to SSBs.

Exposure of M059K cells to ^58^Fe ions using the same experimental conditions shows higher yields of DSBs than X-rays after HTL, but interestingly no detectable modulation after analysis using LTL. This result indicates that the yields of tlDSBs approach zero in M059K cells exposed to ^58^Fe ions.

Differences in the effectiveness at different endpoints between high and low LET radiations are conveniently compared by defining the relative biological effectiveness (RBE) as the quotient of the doses required for equal effect after exposure to X-rays and the test radiation modality. In the experiment shown in Figure 1A and because of the practically linear dose-yield curves measured, this parameter can be determined in an effect-independent manner using the slopes of the resulting straight lines. Notably, widely different RBE values for ^58^Fe versus X-ray exposure are calculated when using as basis the results obtained after HTL (reflecting the sum of prDSBs + tlDSBs) versus LTL (reflecting exclusively prDSBs). Specifically, a value of RBE_HTL_ = 1.12 is calculated using the slopes of the X-ray and ^58^Fe dose–response curves after HTL, while a value of RBE_LTL_ = 1.91 is obtained from the corresponding LTL data.

The response to ^58^Fe noted above is reproduced in the DNA-PKcs deficient counterpart of M059K, the M059J cells [25,26] (Figure 1B). The X-ray data reproduce again earlier findings [17,18] and show that 45% more DSBs are detected after HTL as compared to LTL. On the other hand, after exposure to ^58^Fe, similar DSB yields are obtained after HTL and LTL. As a result RBE_HTL_ = 1.39 and RBE_LTL_ = 2.70 are calculated for the induction of DSBs in M059J cells.

We conclude that in a subgroup of cell lines, examples of which are M059K, and M059J, a contribution of TLSLs to excess DSB formation (tlDSBs) is marginal after exposure to ^58^Fe.

### Detectable formation of TLSL-dependent DSBs in some cell lines after exposure to ^58^Fe

We noted before that the contribution of TLSL to DSBs is cell line specific [17,18] and that this cell line specificity is also detectable after exposure to neutrons [19]. We explored therefore whether this also holds for exposures to ^58^Fe.

**Figure 2:**
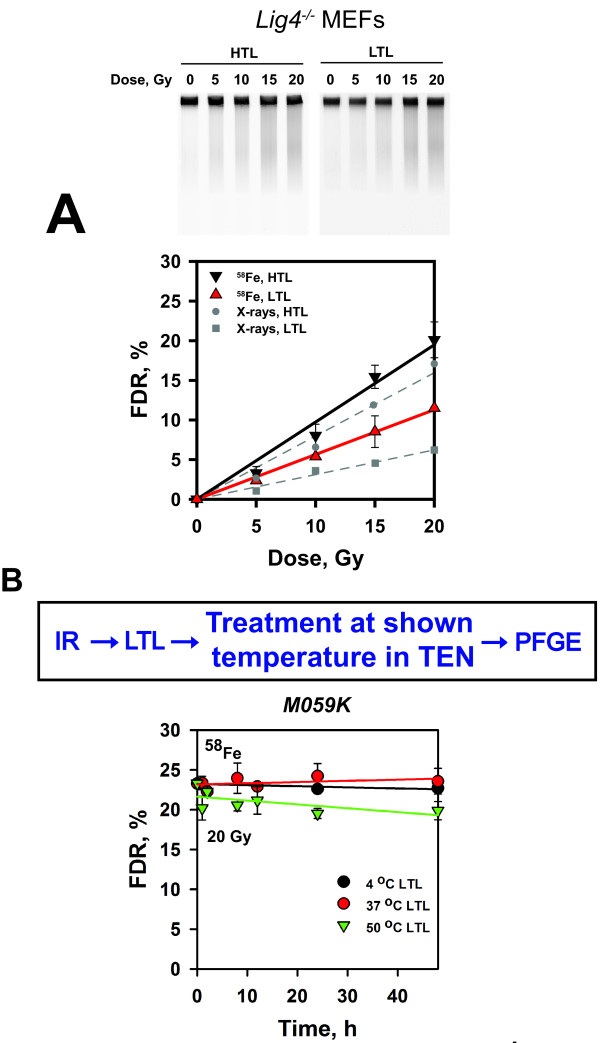
**Induction of DSBs and TLSL evolution in cells exposed to **^**58**^**Fe ions.** (**A**) Exponentially growing *Lig4*^*-/-*^ MEFs were irradiated and analyzed as described in Figure 1. Data represent the calculated average and standard deviation from 4 determinations (agarose blocks) in one experiment. (**B**) Exponentially growing M059K cells were embedded in agarose blocks and exposed to 20 Gy of 1 GeV accelerated ^58^Fe ions. Irradiated blocks were lysed by LTL and incubated in TEN buffer at 4, 37 and 50°C for the indicated periods of time. Cells were analyzed by PFGE. Data represent the calculated average and standard deviation from 4 determinations in one experiment.

The results summarized in Figure 2A indicate a decrease by 63% in the number of DSBs after LTL as compared to HTL in *Lig4*^*-/-*^ MEFs exposed to X-rays. Yet, a decrease by 39% is also registered after exposure of the same cells to ^58^Fe indicating a significant contribution of TLSLs to the formation of DSBs. Here, an RBE_HTL_ = 1.12 and an RBE_LTL_ = 1.83 are calculated for the induction of DSBs after exposure to ^58^Fe.

We conclude that while the contribution of TLSLs to excess DSB formation is reduced after exposure to ^58^Fe, the level of this reduction is cell line dependent.

It may be relevant to mention here that small DNA fragments, undetectable by PFGE, are produced in higher yields after exposure to high, as compared to low, LET radiation. Thus, the yields of DSBs measured after exposure to high LET radiation are likely to be underestimated. However, we consider unlikely that this inherent limitation in the detection of DSBs compromises our conclusions.

### Different yields of TLSL-dependent DSBs after exposure to ^58^Fe of naked DNA and chromatin

To further confirm the absence of TLSL induced DSBs in ^58^Fe exposed M059K cells (Figure 1A), we exposed agarose-embedded cells to 20 Gy and processed them immediately by LTL to obtain agarose-embedded, “naked” DNA in which radiation-induced lesions, including TLSLs, were preserved [18]. In these agarose blocks, TLSL stability can be studied through their contribution to DSB formation after *in-vitro* incubation at different temperatures.

The results summarized in Figure 2B show no significant increase in FDR for incubations in TEN-buffer at temperatures between 4 and 50°C for up to 48 h. Similar experiments carried out with cells exposed to X-rays show large increases in FDR for post-lysis incubations at temperatures above 20°C [18]. The lack of excess DSB formation following incubation at high temperatures of DNA from ^58^Fe-exposed M059K cells is in-line with the similar dose–response curves shown in Figure 1A following HTL and LTL.

Collectively, the above results suggest that cell line specific biochemical parameters contribute to the generation of tlDSBs, even after exposure to high LET radiation. To begin characterizing parameters defining this effect, we used LTL to lyse non-irradiated M059K cells and exposed the resulting agarose-embedded “naked” DNA to 5 Gy of ^62^Ni. In this way, a variety of lesions, including TLSLs, are generated in naked DNA kept in a defined buffer. This condition is biochemically better characterized than irradiation of chromatin organized DNA in the cellular environment.

**Figure 3:**
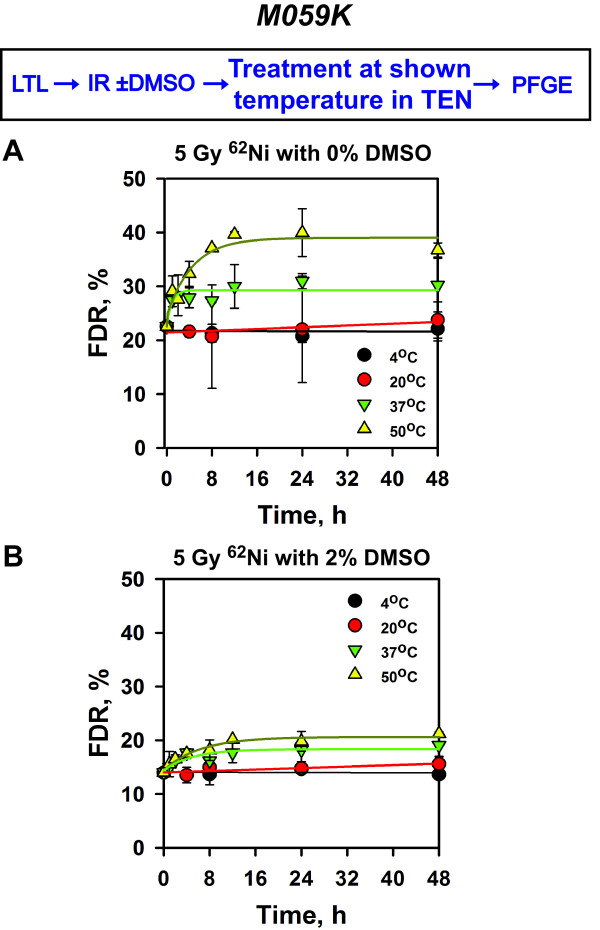
**Induction of TLSL in naked DNA exposed to **^**62**^**Ni ions is suppressed by DMSO.** Exponentially growing M059K cells were embedded in agarose and subjected to LTL. Generated “naked” DNA was exposed to 5 Gy of 1 GeV accelerated ^62^Ni ions in the presence of 0% (**A**) or 2% (**B**) DMSO. Subsequently, agarose blocks were incubated in TEN buffer at different temperatures for the indicated periods of time and analyzed by PFGE. Data represent the calculated average and standard deviation from 4 determinations (agarose blocks) in one experiment.

Agarose blocks generated in this manner were transferred to TEN buffer and after irradiation were incubated at different temperatures for different periods of time. The results obtained are summarized in Figure 3A and show that ^62^Ni ions generate TLSLs in “naked” DNA that readily convert to DSBs after incubation at temperatures between 37 – 50°C, with kinetics similar to that measured after exposure to X-rays [18]. Thus, DNA organization is a key determinant of the chemical characteristics and the associated thermal stability of tlDSBs not only after exposure to low LET but also after exposure to high LET radiation.

We inquired whether indirect radiation effects by water radical production underpin the TLSL-dependent formation of excess DSBs after incubation at high temperatures. For this purpose we carried out the “naked”-DNA experiment described above in the presence of 2% DMSO, an effective scavenger of ^•^OH radicals. The results summarized in Figure 3B indicate that when irradiation of naked DNA is carried out in the presence of DMSO, subsequent incubation at high temperatures reduces the yields of excess DSBs generated, pointing to a contribution of ^•^OH in the production of TLSLs. We conclude that ^•^OH has an essential contribution to the generation of tlDSBs when irradiating “naked” DNA. This result cannot be directly extended to cell irradiation because the chromatin organization of the DNA is protecting it from ^•^OH attacks.

### Processing of DSBs by NHEJ is slower after exposure to HI than X-rays

**Figure 4:**
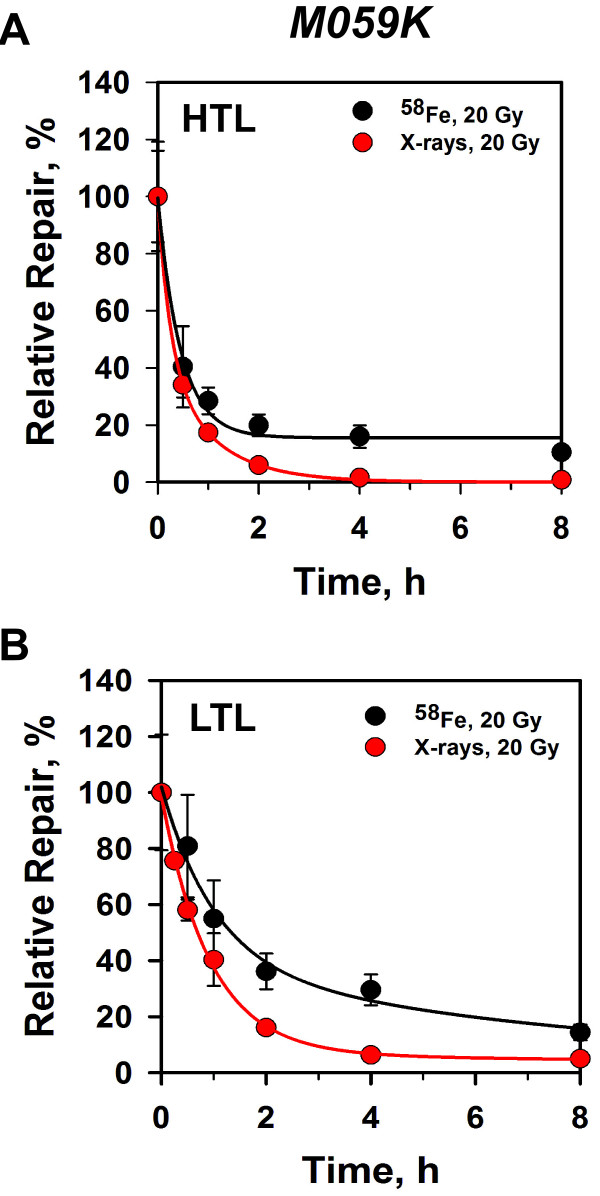
**DSB repair kinetics of human M059K cells exposed to **^**58**^**Fe ions or X-rays.** (**A**) Exponentially growing M059K cells were exposed to 20 Gy of X-rays or ^58^Fe ions and returned to standard incubation conditions for repair. Agarose blocks were prepared and lysed by HTL (**A**) or LTL (**B**) before processing by PFGE. Plotted is relative repair as a function of repair time. To calculate relative repair, FDR at different time points was divided by the FDR measured at the 0 h time point (this value was obtained from the dose response curve). Data represent the calculated average and standard deviation from 4 determinations in one experiment.

In wild-type M059K cells, total DSBs (analyzed by HTL) induced by 1GeV/amu ^58^Fe are repaired with nearly the same efficiency as those induced by X-rays (Figure 4A); however, more unrepaired DSBs are detected between 2 – 8 h after exposure to HI. Within statistical variation, a similar response is also observed when DSB repair kinetics is measured by LTL to specifically assay for prDSBs (Figure 4B). Similar overall trends are also obtained when analyzing wild type MEFs exposed to ^62^Ni ions (Additional file 1: Figure S1).

**Figure 5:**
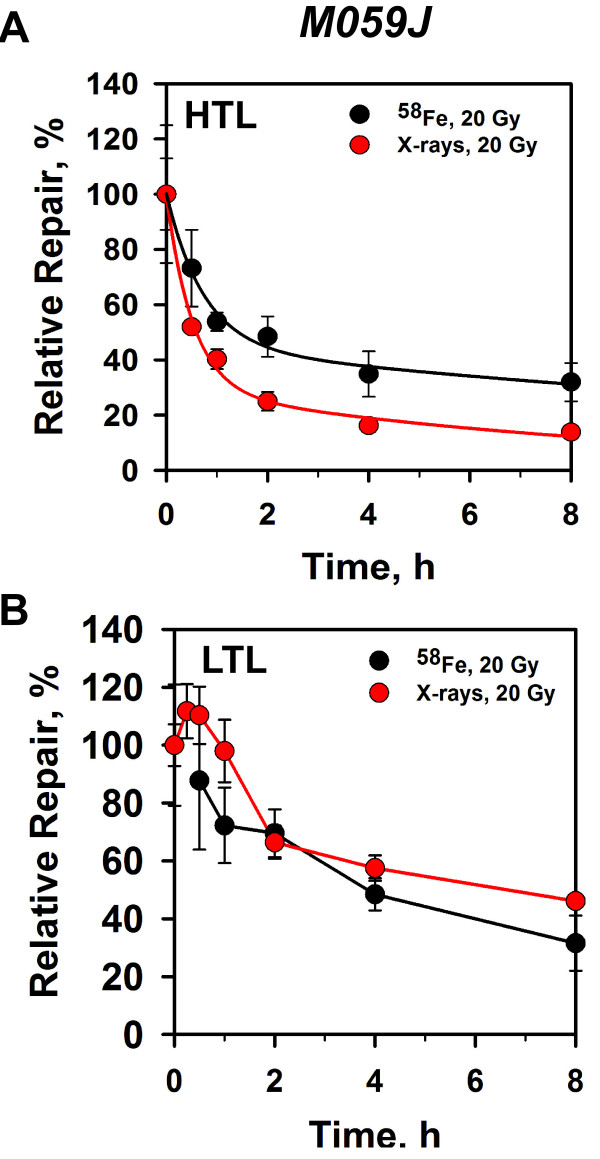
**DSB repair kinetics of human M059J cells exposed to **^**58**^**Fe ions or X-rays.** Other details as in Figure 4.

In *DNA-PKcs* deficient M059J cells (Figure 5A), where D-NHEJ is defective and repair of DSBs is mainly mediated by B-NHEJ [27-29], repair of ^58^Fe-induced total DSBs is compromised slightly stronger than in M059K cells. Thus, the increased DSB complexity of ^58^Fe generated DSBs appears to compromise processing by B-NHEJ to a greater extent than processing by D-NHEJ.

When DSB repair kinetics is measured in M059J cells using LTL to focus analysis on prDSBs and tlDSBs forming during repair, complex kinetics is observed after exposure to X-rays (Figure 5B): The load of DSBs rises at early times and decays subsequently. This structure derives from the fact that the kinetics reflects not only the processing of prDBSs by B-NHEJ, but also the gradual development and subsequent processing of tlDSBs (see [18] for a more in-depth analysis of the components involved). It is not detectable in repair proficient M059K cells, either owing to the higher efficiency of DSB repair, or to differences in the kinetics of tlDSB production.

Repair kinetics after exposure to ^58^Fe ions lacks this structure, in line with the observation that only few tlDSBs are produced in M059J cells after HI radiation (see Figure 1B). We do not have an explanation why similar levels of residual damage is observed after 2 h in cells exposed to X-rays and ^58^Fe when using LTL, but this may reflect analysis artifacts. These may originate from shifts in the yields of prDSBs and tlDSBs with increasing LET, as well as from the normalization applied (to show percent of initial damage). Qualitatively similar results are also obtained after exposure of *Lig4*^*-/-*^ MEFs to ^62^Ni ions (Additional file 2: Figure S2). However, with these cells and probably as a consequence of the significant induction of tlDSBs after exposure to HI (Figure 2A), repair of ^62^Ni induced DSBs is compromised as compared to X-rays when analyzed by LTL (Additional file 2: Figure S2B).

**Figure 6:**
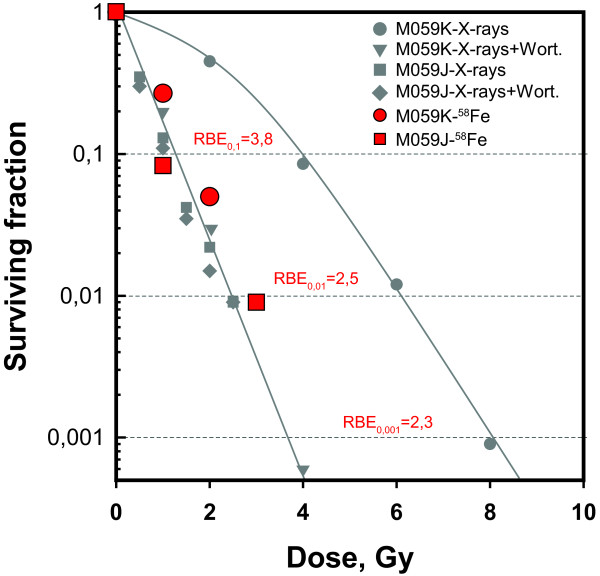
**Survival curves of logarithmically growing M059K and M059J cells as measured by colony formation after exposure to 1 GeV **^**58**^**Fe ions (red symbols).** Shown (gray symbols) for comparison are also results of experiments previously reported [24] with these cells after exposure to X-rays in the presence or absence of 20 μM wortmannin. The figure also shows RBE values calculated at the indicated levels of cell survival. The lines drawn follow the early data points [24] and are given as basis for comparisons with the results generated with ^58^Fe.

In an effort to connect the above observations on the induction and repair of different forms of DSBs with the cell inactivation potential of HI, cell survival was determined. The results obtained with M059K and M059J cells are summarized in Figure 6. Shown in the figure for comparison are also results previously reported with the same cell lines after exposure to X-rays, in the presence or absence of 10 μM wortmannin to inhibit D-NHEJ [24]. M059K cells exposed to X-rays show the typical dose response, characterized by a shoulder at low radiation doses followed by an exponential region at higher doses. Wortmannin-induced inhibition of D-NHEJ strongly sensitizes M059K cells to X-rays and leads to an exponential survival curve. Notably, M059K cells exposed to ^58^Fe ions show cell survival practically indistinguishable from that of wortmannin-treated cells. From these results RBEs of 3.8, 2.5, and 2.3 are calculated at surviving fractions of 0.1, 0.01, and 0.001, respectively.

M059J cells, as a result of their deficiency in D-NHEJ, are intrinsically highly radiosensitive and wortmannin has no further radiosensitizing effect. Notably, ^58^Fe ions are killing these cells with efficiency practically indistinguishable from that of X-rays, which leads to RBEs of approximately 1. This observation is in line with earlier reports pointing to a D-NHEJ proficiency requirement for high LET mediated radiosensitization [30-33].

## Discussion

There is evidence for the induction by IR of thermally labile DNA lesions, which contribute to DSB formation (tlDSBs), albeit in a delayed manner, even in cells maintained under physiological temperatures (see Introduction). As a result of this delayed formation, the total load of DSBs generated in an irradiated cell (tDBSs) will be the sum of those induced promptly, i.e. those present immediately after irradiation (prDBSs), and those generated within a non-DSB-CDS by the conversion of a TLSL to a SSB (tlDBSs); thus, tDSBs = prDBSs + tlDBSs. It is not known whether prDBSs and tlDBSs are detected and processed by the cell with the same efficiency and, actually, arguments can be developed why this may not be the case [17-19]. If cells detect and process differently prDBSs and tlDBSs, it is likely that their biological consequences will also be different.

Experimentally, the yields of prDBSs can be determined by lysing cells immediately after irradiation using low temperature (0 – 4°C) lysis protocols (LTL), whereas the standard 50°C lysis allows determination of tDBSs. The difference between tDBSs – prDBSs yields gives then estimates regarding the yields of tlDBSs. There is evidence that IR induces a spectrum of TLSLs with different levels of chemical and thermal stability [18]. This raises the question how to determine the biologically relevant subset of tlDBSs, i.e. the subset that also converts to a DSB in cells maintained under physiological conditions. There are at present no established methods allowing the reliable determination of the biologically relevant subset of tlDBSs. However, as a first approximation, we assume that conversion of TLSLs to breaks is similar in cells maintained at 37°C and cells analyzed by lysing at 50°C immediately after exposure to IR [18].

Using the above outlined conceptual and experimental background we investigate here how the yields of prDBSs and tlDBSs change in cells exposed to HI. The results presented in the previous section extend trends previously reported for neutrons [19] and confirm a strong, inverse LET dependence of the yields of prDBSs and tlDBSs. Specifically, while exposure to HI causes a strong reduction in the yields of tlDBSs as compared to X-rays, it causes a strong increase in the yields of prDBSs. These opposing effects partly compensate each other and as a result the yield of tDSBs changes only modestly with increasing LET. This is in line with the observation that RBEs close to 1 are frequently measured for the induction of DSBs [34]. Notably, our results demonstrate that when prDBSs are specifically detected by LTL protocols, much higher RBE values are measured that are approaching those obtained for cell survival (Figure 6). This is a potentially highly significant observation that warrants further investigations.

Notably, the RBE values for DSB-induction after exposure to high LET radiation, as measured by γ-H2AX foci formation in diverse cell lines, is also very close to one ([34] and references therein). This is significant as it shows, in line with our earlier work [18], that the load of DSBs the cell ultimately “sees” is close to that measured by HTL. If cells were only detecting prDBSs, as it is often assumed, two to three times more DSBs (i.e. γ-H2AX foci) would have been expected after exposure to high LET radiation than after low LET radiation. On the other hand, it also demonstrates that the γ-H2AX marking of DSBs does not differentiate levels of DSB complexity.

The increase in prDSBs observed with increasing LET can be explained by the expected increase in the size of ionization clusters (more ionizations within the same volume) that leads to the generation of higher complexity CDS, i.e. the presence of a higher number of lesions at the site. As a result of this increase in lesion number within a CDS it becomes more likely that prompt SSBs will combine to form a prDSB. Even if TLSLs are present in these CDSs, their subsequent conversion to breaks will remain inconsequential with reference to DSB formation. The chemical reactions that convert a TLSL to a SSB remain uncharacterized, but may include base-catalyzed hydrolysis or oxidation [19].

As noted above, TLSLs are not a uniform chemical entity but rather a spectrum of lesions with different chemical and thermal sensitivities. The probability of their formation from clusters of ionization events and radical attacks, as well as their chemical evolution may be decisively determined by the chemical environment in their immediate vicinity. In this respect, it is likely that the details of DNA organization in chromatin within a CDS, including all participating histone and non-histone proteins, will affect decisively not only the induction of TLSLs but also their evolution to tlDBSs. This theoretically anticipated dependence provides also a first explanation for the surprisingly large differences observed in the yields of tlDBSs among different cell lines both after exposure to high as well as to low LET radiation [17-19]. Furthermore, the large differences in LTL dose response curves among different cell lines contrasts the surprisingly similar HTL dose response curves and points to cell line specific variation in the chemical environment in the vicinity of a clustered ionization hitting the DNA that alters the probability of generation of a prDSB.

While the selection of cell lines used here reflects the intellectual evolution of the TLSL problematic in our work during the past few years, future work will certainly benefit from a hypothesis oriented selection of cell lines and an analysis of TLSL production and evolution after treatments that alter chromatin organization. It is also worth pointing out that the differences between cell lines persist even after exposure of cells to high LET radiation [19] (see above).

The results discussed above demonstrate that the energy deposition pattern of radiation is not the sole determinant of the yields of tlDSBs. Small differences in DNA organization may cause changes in the induction and the subsequent chemical processing of TLSLs and may strongly affect the form of DSBs induced – even after exposure to high LET radiation. Worth noting is also that the differences observed in lesion induction and evolution among cell lines exposed to HI are largely eliminated if “naked” DNA is irradiated instead of cells.

Collectively, the results presented here, as well those published before [17-19], point to an unexplored dimension in the production of DNA damage by IR. Temporal evolution of complex radiation damage to DSBs, and the suggested role of DNA organization in this evolution go beyond current concepts of DNA damage induction and repair [35] and indicate aspects of DDR that warrant further investigations.

It is tempting to speculate that transient, chemical stabilization of TLSLs, may allow repair of SSBs and base damages within a non-DSB CDS, so that subsequent conversion of the TLSL to a DNA break will not cause a DSB. Such agents may find application in radiation protection on earth and in space, as well as in the development of new strategies in radiation oncology [21,36,37].

## Competing interests

The authors declare that they have no competing interests.

## Authors’ contributions

SKS designed and performed the PFGE experiments, analyzed and interpreted the results obtained and contributed to the preparation of the manuscript. ABT designed and performed the cell survival experiments. EM performed cell survival experiments and helped in the preparation of the manuscript. BJ and GTS helped design the irradiations with heavy ions at the GSI and assisted in the interpretation of the results obtained, as well as in the preparation of the manuscript. GI conceived the project, assisted in the experimental design, interpreted the results obtained and wrote the manuscript. All authors read and approved the final manuscript.

## Supplementary Material

Additional file 1: Figure S1 Repair kinetics of exponentially growing wild type MEFs after exposure to 1GeV ^62^Ni ions or X-rays. Other details as in Figure 4.
Click here for file

Additional file 2: Figure S2 Repair kinetics of exponentially growing *Lig4*^*-/-*^ MEFs after exposure to 1GeV ^62^Ni ions or X-rays. Other details as in Figure 4. Click here for file
